# Gut Bacteria Cospeciating with Insects

**DOI:** 10.1371/journal.pbio.0040357

**Published:** 2006-10-10

**Authors:** Liza Gross

With some 1 million species and counting, insects may be the most abundant class of animals living today. Their protective exoskeleton, prolific reproductive rate, and wings help their cause, as do the symbiotic bacteria that inhabit their cells, gut, or body cavity. Endocellular symbionts live inside specialized insect cells and provide essential nutrients for their hosts, which in turn provide suitable habitat for the bacteria. Insect mothers transmit endocellular symbionts to their offspring during egg or embryo development, preserving an intimate bond between host and symbiont that is evident in both species' genomes.

Studies that use genome analysis to infer evolutionary relationships (called phylogenetics) show that the history of insect host genes (or phylogeny) often mirrors that of their endocellular symbiont—indicating a shared evolutionary history, or cospeciation. Unlike endocellular symbionts, gut or body cavity symbionts are vulnerable to displacement or attack by other microbes and appear to have less-exclusive relationships with their hosts, based on reports that host–symbiont phylogenies for termites and alydid stinkbugs do not match. But a new study suggests that not all gut symbionts go for the promiscuous lifestyle. Takahiro Hosokawa, Takema Fukatsu, and colleagues provide the first evidence of cospeciation between a group of gut symbionts and their insect hosts, plataspid stinkbugs. Not only do their phylogenies mirror each other, but the gut symbionts share many of the unique genetic traits typical of endocellular symbionts.

Plataspid stinkbug symbionts live in the bugs' posterior midgut and are vertically transmitted by the mother in symbiont “capsules.” When the female lays eggs, small, brown symbiont-filled capsules always appear under the egg mass. Nymph hatchlings ingest symbionts from the capsule.

Hosokawa et al. collected 12 populations of stinkbugs, representing three genera and seven species, from several locations in Japan. (Four species were used in the experiments.) All females had the same three-compartment midgut, which had been previously described in two other species: one section contains the symbionts (called the thin crypt-bearing midgut, or TCM), another secretes webbing that embeds the symbionts into the capsules, and a third produces the shell that encases the capsule. All the females also codeposited capsules and egg masses. (Males have only the TCM.)

After removing the TCM from adult females, the researchers analyzed the DNA of the resident bacteria—focusing on a ribosomal RNA gene called 16S rRNA often used to identify bacteria—and found that each bacterial species was associated with a different stinkbug species. Using the 16S rRNA sequences to infer the bacteria's evolutionary origins, they discovered that the sequences didn't match any other bacterial sequences in the databases—they fell into their own class of Proteobacteria. Interestingly, however, the symbionts did form a sister group—indicating evolutionary kinship—with the well-characterized obligate endosymbiont (Buchnera aphidocola) of aphids.

Given the phylogenetic similarity between the stinkbug symbionts and Buchnera, the researchers wondered whether their biology might be similar as well. They divided egg masses into two groups and deprived one group of capsules to generate sibling populations with and without gut symbionts. Adults lacking symbionts showed developmental delays, grew smaller, failed to copulate or reproduce, and died prematurely. Like aphids depend on their endosymbionts, plataspid stinkbugs depend on their gut symbionts to survive—how they do this, however, will be interesting to discover. Like Buchnera, the gut endosymbionts also appear to have co-evolved with their host. The phylogenetic tree of the stinkbugs, the researchers found, “perfectly agreed” with the phylogenetic relationships of the gut symbionts. Maternal transmission of the symbiont capsule provides a means of stable transmission, but other factors such as physiological compatibility may come into play.

The symbiotic lifestyle appears to have shaped the genome evolution of endocellular symbionts, which have a small genome, a high percentage of A and T nucleotides in their DNA, and accelerated molecular evolution. Whether these genetic traits arose from population genetic forces—for example, small population size and bottlenecks—or from some aspect of the endocellular environment has been a matter of dispute. Hosokawa et al. found the same “peculiar” genetic patterns in the gut symbionts, lending support to the population genetic hypothesis. They named these gut symbionts “Candidatus Ishikawaella capsulata,” in honor of Hajime Ishikawa, a pioneer in the molecular study of symbiosis, who recently passed away.

How the symbiont capsule evolved remains an open, and intriguing, question. With some 530 species and 56 genera in the Plataspidae family, researchers have their work cut out for them as they survey the lineages for a stinkbug without a capsule. But with this unique plataspid stinkbug system, they will be well equipped to study insect symbiosis and its influence on genome evolution.

## 

**Figure:**
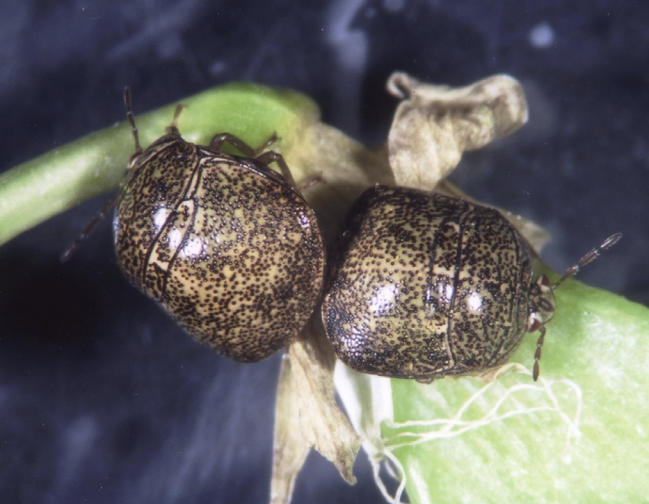
A mating pair of the Japanese common plataspid stinkbug Megacopta punctatissima.

